# Supplementary material for: Clonal Dispersal of Cryptococcus gattii VGII in an Endemic Region of Cryptococcosis in Colombia
Source: J Fungi (Basel). 2019 Apr 15;5(2):32. doi: 10.3390/jof5020032 (PMC6616963; doi:10.3390/jof5020032)
Supplement: Supplementary file 1 [file jof-05-00032-s001.pdf]

Supplementary tables

Table 1. General information, mating type, allele types, and sequence types of additional *Cryptococcus gattii* molecular type VGII isolates included in the MLST analysis [9,22,23,32–34].

**Strain number**	**Other number**	**Country** [a]	**Source** [b]	**Mating type**	***CAP59***	***GPD1***	**IGS1**	***LAC1***	***PLB1***	***SOD1***	***URA5***	**ST**
WM 178	CBS 10082	Aus	Clin	alpha	1	17	16	16	14	19	7	21
WM 04.72	PWQ 314	Aus	Clin	alpha	3	2	6	7	1	18	5	33
WM 08.309	SOLIDAGO	Aus	Vet	alpha	27	6	4	4	1	43	7	48
WM 08.311	Jackson	Aus	Vet	alpha	3	16	15	4	9	23	2	5
WM 09.94	1009	Aus	Vet	**a**	14	21	27	28	27	46	2	38
CBS11260		Bra	Vet	alpha	68	6	82	4	16	94	6	248
CDC-3225		Bra	ND	alpha	21	6	25	4	2	27	7	264
CDC-3234		Bra	ND	alpha	33	6	25	29	1	1	1	137
CDC-3243		Bra	ND	alpha	2	6	16	4	16	4	2	283
ICB180		Bra	Env	alpha	10	6	16	4	16	20	2	185
ICB184		Bra	Env	alpha	14	6	15	4	2	15	2	124
WM 04.84	LA 337	Bra	Clin	alpha	5	30	25	4	16	14	6	34
WM 05.419	LMM 293	Bra	Clin	alpha	14	6	39	27	29	25	9	39
WM 05.452	LMM 417	Bra	Clin	alpha	2	27	15	4	1	8	7	16
WM 05.456	LMM 498	Bra	Env	alpha	2	6	10	4	2	11	7	17
WM 05.457	LMM 504	Bra	Clin	**a**	2	27	31	4	1	8	7	19
WM 05.461	LMM 567	Bra	Clin	alpha	8	6	41	4	1	16	6	14
WM 05.462	LMM 574	Bra	Clin	alpha	8	6	36	4	1	16	6	15
WM 05.525	LMM 631	Bra	Clin	alpha	8	6	42	25	1	24	7	41
WM 05.527	LMM 634	Bra	Clin	alpha	34	6	9	4	16	9	2	28
WM 05.528	LMM 843	Bra	Clin	alpha	4	22	26	4	27	7	3	40

WM 05.529	LMM 855	Bra	Clin	alpha	2	32	25	30	1	13	7	27
WM 05.530	LMM 860	Bra	Clin	alpha	1	1	31	21	27	7	4	13
WM 05.533	LMM 892	Bra	Clin	alpha	10	6	16	30	16	20	2	11
WM 05.536	LMM 638	Bra	Clin	alpha	2	6	4	4	16	4	2	9
WM 05.545	LMM 817	Bra	Clin	alpha	13	6	10	26	18	17	8	24
WM 05.546	LMM 818	Bra	Clin	alpha	13	6	25	26	18	17	8	23
WM 05.547	LMM 819	Bra	Clin	alpha	13	6	26	26	18	17	8	22
WM 04.78	H0058-I-762	Col	Clin	alpha	4	6	6	4	18	22	2	31
WM 05.274	H0058-I-1442	Col	Clin	alpha	12	29	6	21	22	21	2	29
WM 05.339	H0058-I-1648	Col	Env	alpha	1	6	25	4	18	12	10	43
WM 05.76	AV54S; CBS 10089	Gre	Clin	alpha	8	16	35	4	2	3	7	35
WM 05.77	AV55; CBS 10090	Gre	Clin	**a**	2	27	10	4	1	8	7	18
WM 04.75	MC-S-115	Tha	Clin	alpha	2	6	32	4	2	15	2	30
WM 06.8	LA 43; CBS 8684	Uru	Env	alpha	7	6	32	21	25	2	2	3
B7390		USA	Clin	alpha	30	6	15	4	1	15	2	49
B7432		USA	Clin	ND	4	6	15	4	1	15	2	6
B8973	2010721506	USA	Clin	alpha	2	25	4	21	9	8	7	50
WM 1851	LA 387	Ven	Clin	alpha	22	21	40	4	14	26	2	45
WM 06.12	LA 381	Ven	Clin	alpha	2	6	31	4	18	12	10	37

[a] Isolates recovered in Aus: Australia, Bra: Brazil, Col: Colombia, Gre: Greece, Tha: Thailand, Uru: Uruguay, USA: United States of America, and Ven: Venezuela.; [b] Clin: Clinical; Env: Environmental; Vet: Veterinary.; ND: No determined.

Table 2. Organ burden of mice infected with *Cryptococcus gattii* isolates molecular type VGII.

	Organ burden (log 10 CFU/g)		
**Strain**	**Brain**	**Lung**	**Spleen**
ENV152 (VGIIa)	6.75 ± 1.09	5.56 ± 0.65	3.97 ± 2.20
H0058-I-1511	7.69 ± 0.45	6.88 ± 0.52	4.78 ± 2.57
H0058-I-2792	7.37 ± 0.48	7.24 ± 0.33	5.20 ± 0.68
RB28 (VGIIb)	7.26 ± 0.36	6.98 ± 0.49	5.69 ± 1.04
H0058-I-357	8.69 ± 0.68	7.47 ± 3.23	6.41 ± 1.23
H0058-I-3030	8.03 ± 4.27	6.88 ± 3.76	3.18 ± 1.55
Control WM 198	7.49 ± 0.77	7.03 ± 0.39	4.84 ± 0.80
Saline	0	0	0
